# Spanish translation and validation of the Music-Related Quality of Life Questionnaire (MuRQoL) in postlingually deaf cochlear implant users

**DOI:** 10.1007/s00405-024-08628-7

**Published:** 2024-04-26

**Authors:** Alejandro Zuazua-Gonzalez, Miryam Calvino, Álvaro Postigo, Carlos Domingo, Javier Gavilán, Luis Lassaletta

**Affiliations:** 1 Department of Otorhinolaryngology and Head and Neck Surgery, Hospital Universitario Infanta Leonor, Madrid, Spain; 2 Department of Otorhinolaryngology, Hospital Universitario La Paz. IdiPAZ Research Institute. Universidad Autónoma de Madrid, Madrid, Spain; 3 Biomedical Research Networking Centre on Rare Diseases (CIBERER), Institute of Health Carlos III (CIBERER-U761), Madrid, Spain; 4 Department of Psychology, Universidad de Oviedo, Oviedo, Spain

**Keywords:** Cochlear implant, Music, Quality of life, Questionnaire, Validity, Meludia

## Abstract

**Purpose:**

The aim of this study was to translate and validate the “Music-Related Quality of Life Questionnaire” into Spanish (sMuRQoL) and assess its convergent validity and discriminative capacity by comparing its scores with the outcomes of the musical perception test Meludia.

**Methods:**

The sMuRQoL was completed by 129 patients: 55 cochlear implant (CI) users and 74 normal hearing (NH) individuals. Conducted in this study were an exploratory factor analysis, an evaluation of internal consistency, an assessment of score stability through test–retest reliability, a comparison of sMuRQoL scores between CI users and NH individuals and an examination of potential evidence of convergent validity and discriminative capacity of sMuRQoL in relation to other tools. This involved the comparison of the questionnaire scores with the Meludia outcomes.

**Results:**

The sMuRQoL demonstrated a two-dimensional structure. All the dimensions displayed high internal consistency (α = 0.879–0.945) and score stability (*ICC* = 0.890–0.942). There were significant differences in the Frequency test between NH and CI users (*d* = 1.19–1.45). There’s evidence of convergent validity between the scores of the Frequency test and the results of Meludia (*r* = 0.242–0.645). Additionally, the Frequency test demonstrate a good discriminative capacity to identify patients with poorer musical perception.

**Conclusions:**

The sMuRQoL is a reliable questionnaire, with adequate evidence of validity based on internal structure. This study provides an accessible, cost-effective, and quick-to-administer instrument in Spanish, optimizing available healthcare resources and bringing us closer to the patient needs.

## Introduction

### Music and cochlear implants

With the contemporary cochlear implants (CIs), users of all ages can achieve high levels of speech perception [1]. Nonetheless, musical perception is particularly challenging for these individuals. This challenge arises from biological constraints imposed by their auditory pathology, and the spectral intricacies of music, as opposed to speech, makes music perception a demanding task [2].

CI users employ different strategies to deal with deficits in musical perception. While many children or adolescents with congenital or prelingual hearing loss view music positively [3] and are actively involved in musical activities [4], adult with CI tend to avoid music due to its perceived unpleasantness [5, 6]. This issue can affect the overall quality of life for CI users, as music is present in countless social and cultural contexts in daily life [7].

In two studies carried out by Lassaletta et al., a positive correlation was found between the perceived quality of music and overall quality of life. The studies indicated that users who spend more time listening to music after implantation tend to exhibit higher quality of life scores [6, 8]. These findings further support the importance of music.

In this context, various programs aimed at training and evaluating musical perception following cochlear implantation have been developed [9–14], as musical training appears to have significant and lasting effects, particularly when initiated at an early developmental stage [9]. This training can enhance musical perception mediated by CIs. However, many of the studies conducted so far involve in-person musical training programs that are time-consuming and less accessible. Online resources are a growing potential resource. In Jiam’s study [11], timbre identification scores improved to a greater extent after online musical training. Smith et al. [13] found that scores on post-training diagnostic tests using self-administered musical training software were better compared to pre-training scores, with significant improvements observed in the perception of musical patterns.

Musical training with platform may be used as supplement to the standard speech therapy rehabilitation [15] in patients following cochlear implantation. It serves as an adjunctive therapy training program, rather than replacing traditional rehabilitation methods. However, a “Gold Standard” for evaluating musical experience has not been adopted [16], which would allow for the individualization of auditory training for each patient.

The Meludia platform (Meludia, Paris, FR) is among the various tools designed for musical training. A recent study has proposed Meludia as a suitable means for objectively evaluating musical perception and as a tool for musical training in CI users over 6 years old [17]. However, access to Meludia and similar platforms is not widespread across all Centers where CI surgeries and rehabilitation are conducted. Consequently, alternative tools such as questionnaires play a crucial role in assessing the perception and enjoyment of music in these patients. The Munich Music Questionnaire (MUMU) and the Music Related Quality of Life Questionnaire (MuRQoL) have been validated for evaluating these aspects [7].

### Need for the validation of MuRQoL to Spanish

This questionnaire, originally in English, is highly comprehensive and evaluates the impact of music on quality of life across two dimensions: the frequency of perception and musical engagement (questions 1–18, part I), as well as musical importance (questions 1–18, part II). It is a questionnaire that can be easily administered during a routine visit to the Clinic. Recently, versions in Turkish [18] and Italian [19] have been validated. Spanish is the world's second-most spoken native language after Mandarin Chinese. To the best of our knowledge, the MuRQoL has not been validated in Spanish before.

### Objectives

The primary goal of this study was to translate and validate a Spanish version of the MuRQoL (sMuRQoL), making it widely available in clinical settings and allowing us to better understand the needs of adult CI users.

In the original MuRQoL’s instructions for use [7], it is also suggested that, for a comprehensive assessment of music, it is preferable to combine the MuRQoL questionnaire with music perception tests. We deemed it pertinent to explore whether this subjective level of musical perception (high or low) corresponded to outcomes (adequate or insufficient) in objective musical training tasks on the Meludia platform. In this manner the sMuRQoL could be utilized both for its initial purpose—assessing Music-Related Quality of Life- and for identifying individuals who may be “at risk” for poorer musical perception. This application of the MuRQoL was suggested in the original article [7]. Consequently, we could identify these patients much earlier and introduce them to musical training programs following cochlear implantation. Hence, a secondary objective was to gather evidence related to the convergent validity and discriminative capacity concerning musical perception using the sMuRQoL. This was achieved by comparing the scores of the sMuRQoL’s Frequency Test with the outcomes of the same patients in the objective musical perception test, Meludia.

## Materials and methods

### Translation of MuRQoL

The MuRQoL consists of two sections, each comprising 18 questions. The first part -Frequency Test- examines subjective musical perception abilities, while the second part -Importance Test- evaluates the perceived importance of musical perception and attitudes towards involvement with music [7]. Responses are rated on a 5-point Likert scale ranging from 1 (never) to 5 (always). The scores for both frequency and importance of each item can be compared to create a visual representation of how each of the 18 music perception/engagement tasks impacts the quality of life of individual CI users. When music is regarded as important but the frequency score for the perception or engagement subscale (or the overall frequency scale score) is low, it suggests that music may have a significant negative effect on quality of life. A patient scoring within this critical range for a music perception/engagement item indicates a need for music training, targeting specific musical aspects. This approach enables the MuRQoL measure to serve as a diagnostic tool for identifying individuals requiring music-related support and for guiding music training efforts.

The items and the overall score of the sMuRQoL underwent validation based on the psychometric criteria of classical test theory [20]. The translation of the “Music Related Quality of Life Questionnaire” into a Spanish version was carried out with the approval of the author of the original questionnaire. This involved the translation of the MuRQoL into Spanish by bilingual individuals, followed by a back-translation from Spanish to English conducted by professional translators. The back-translation was compared with the original questionnaire and the final version of the sMuRQoL (see Supplementary material) was approved.

### Meludia

Currently, Meludia is available in up to 20 languages, some of them are English, French, German, Italian or Spanish. For this study, the Spanish version of the Meludia Discovery module [21] was employed for assessment, comprising five tasks, each with five levels of difficulty, totaling 25 exercises:

- Rhythm: Determining the number of percussive hits heard.
- Spatialization: Identifying whether the second note is higher or lower.
- Melody: Recognizing whether the melody is ascending or descending.
- Stable/unstable: Assessing whether the sound feels stable or unstable.
- Density: Estimating how many sounds are played simultaneously—whether it’s one or many.

Before the assessment, a Spanish version program demonstration was provided to all participants in a quiet setting. NH subjects used headphones connected to a laptop.

CI users, utilized the direct audio input (DAI) cable to ensure that assessment hearing was conducted solely through the CI, eliminating input from the contralateral ear in cases of residual hearing. Bilaterally implanted participants were assessed with both implants simultaneously.

For each exercise, Meludia generates a numerical score ranging from 0 to 3. A score of 0 signifies an incomplete attempt, while 3 indicates the fastest and most accurate performance on the exercise. Participants were allowed to restart each level up to four times. If a level remained incomplete after four attempts, that task was considered unfinished. The program proceeded to the next exercise when the 5 levels of each task were completed or unfinished before.

The tasks were presented in the following sequence: Rhythm, Spatialization, Melody, Stable/unstable and Density (See Supplementary material for an example of Meludia Testing Procedure). The Meludia testing procedure require an average time of 60 min to be completed.

### Participants

The minimum sample size was calculated considering the maximum variance of the original study scale [7] and considering a 95% confidence interval. Therefore, with the variance of the original study being around 16 and considering a 95% confidence interval, a minimum sample size of 123 (122.93) individuals is estimated to be necessary.

We recruited two groups of participants: 55 individuals with CI and 74 subjects with NH. The group of CI users consisted of postlingually deaf individuals aged ≥ 17 years. All of them had been implanted with a MEDEL CI system (MED-EL GmbH, Innsbruck, Austria), either unilaterally or bilaterally. Additionally, they had a minimum of 10 active electrodes and had maintained a stable fitting (at least one year without any changes in the fitting of the CI).

The individuals with NH were selected from the Otolaryngology Outpatient clinics, specifically among those seeking consultation for conditions unrelated to hearing. The participants in this group did not have a formal musical academic background, and their ages were matched to those in the CI user group. Additionally, they underwent an audiometric evaluation and only those with a PTA < 30 dB were included in the study.

All participants were proficient in Spanish and did not have simultaneous visual or cognitive impairments that could interfere with task performance.

### Ethics committee

The design of the study was approved by the local Ethics Committee (approval number HULP PI-4447) and was registered in ClinicalTrials.gov (identifier NCT05319678). No adverse events were reported during the study.

The research was conducted in accordance with the principles of the Declaration of Helsinki [22] and informed consent was obtained from each participant.

### Data collection

The final version of the sMuRQoL was completed firstly in-person under supervision during Otolaryngology consultations, both for NH subjects and CI users. For those subjects who took the test in two occasions − 58 NH subjects and 37 CI users-, some answered it again in-person, while others did so through an online self-administered form.

### Data analysis

Only fully completed questionnaires with fewer than 3 N/A responses were considered. For those who underwent the test–retest, only patients who completed it within the specific time frame (15 days after the initial assessment) were selected.

1. Firstly, descriptive statistics (mean, standard deviation, skewness, and kurtosis) of the instrument’s items were analyzed. The discrimination indices of the items (corrected item-test correlation) were analyzed, considering them adequate when they were above 0.20 [23].
2. The internal structure of the sMuRQoL was analyzed through an Exploratory Factor Analysis (EFA). This procedure was conducted for both the first part of the questionnaire—Frequency test—and the second part—Importance test. As all items exhibited suitable values of skewness and kurtosis (within a range of ± 1), and the number of response alternatives was five, the EFA was conducted on the Pearson correlation matrix [24]. Unweighted Least Square (ULS) was employed as the estimation method. The KMO index and Bartlett’s test of sphericity were used to assess the data’s suitability for the EFA. A value of KMO above 0.70 and a statistically significant Bartlett’s test value (*p* < 0.05) indicate that the data are suitable for factor analysis. The Measure of Sampling Adequacy (MSA) was also calculated, where values of MSA below 0.50 suggest that the item does not measure the same domain as the remaining items in the pool, and so it should be removed. Promin was used as the rotation method since it deals with oblique factors. The percentage of explained variance was assessed and fit indices such as Comparative Fit Index (CFI), and Root Mean Square of Residuals (RMSR) were used, establishing a good fit when CFI > 0.95 and RMSEA < 0.08 [25].
3. The reliability of the sMuRQoL scores were examined. For the study of internal consistency, Cronbach alfa’s coefficient and McDonald’s omega coefficient were used. Additionally, the stability of the scores (test–retest reliability) was studied through the intraclass correlation coefficient. Values above 0.70 indicate good internal consistency and stability of the scores.
4. The mean score was calculated for the frequency and importance tests, as well as the perception and engagement subscales for each group of participants (CI and NH). Similar to the original MuRQoL validation study and based on literature indicating significantly poorer musical performance of adult CI users compared to adults with NH in pitch perception, timbre, and recognition of familiar melodies, the hypothesis was formulated that the Frequency test scores of the sMuRQoL for adults with NH was significantly higher than those of CI users for the overall scale and subscales. Regarding the scores in the Importance test, we hypothesized that no significant differences would be expected between both groups, as there is no evidence of a different level of importance of music between NH subjects and CI users. To study potential differences in sMuRQoL scores between NH subjects and CI users, an independent samples t-test was conducted and Cohen’s *d* was used as the effect size. Effect sizes were categorized as small for values between 0.2 and 0.5, medium for values between 0.5 and 0.8 and largo values for above 0.8 [26].
5. The potential convergent validity of the sMuRQoL in relation to other tools was examined. As the first part of the MuRQoL-Frequency Test- includes a series of questions about individual ability to perceive music—especially items 1–11—, the hypothesis was formulated that those subjects who scored higher in the Frequency scale would also achieve better results in the Meludia Discovery module. In the Importance scale, no differences are expected between groups in the Meludia results since the subjective importance of music for each individual does not confer greater musical perception ability. For this analysis, the Pearson correlation was calculated between the scores of the instrument and the score of the first attempt in each of the five categories of Meludia (Rhythm, Spatialization, Stable/unstable, Melody and Density). Of the 129 participants who completed the sMuRQoL, 79 of them completed Meludia (35 CI users and 44 NH individuals).
6. Finally, ROC curves were calculated to study the discriminative capacity of the sMuRQoL in detecting subjects with heightened musical perception. In the original validation, it was concluded that the MuRQoL has the potential as a tool for pinpointing those who could benefit from musical training. However, this had to be demonstrated in clinical practice and future experience. In this regard, we hypothesized a potential discriminative capacity in the first part of the test (Frequency), and to substantiate this, we used the five Meludia Discovery tasks completed in a single attempt as the “Gold Standard”. The discriminative capacity was assessed using the area under the curve (AUC). Values between 0.5 and 0.6 are considered inadequate, acceptable between 0.6 and 0.8, and about 0.8 are considered quite good.

EFA were conducted using the FACTOR 12.03.02 program [27]. The rest of the statistical analyses were performed using the SPSS v27 (IBM Corp, 2021).

## Results

A final sample of 129 patients was obtained, of which 95 of them (74%) completed the questionnaire on two occasions with a 15-day interval between determinations.

The demographic and clinical data (gender, age, patient type, educational level, time and type of cochlear implantation), as well as the performed instruments, are recorded and summarized in Table 1*.*

**Table 1:** Demographic and clinical data and instruments performed

	Total	CI users	Normal hearing
*N* (%)	129	55 (43)	74 (57)
Gender			
Men, *N* (%)	57 (44)	26 (47)	31 (42)
Women, *N* (%)	72 (56)	29 (53)	43 (58)
Age ± SD (range) (years)	55 ± 15 (17–86)	56 ± 17 (17–86)	54 ± 14 (21–80)
CI duration^a^ ± SD (range) (years)	9 ± 7 (1–21)	9 ± 7 (1–21)	N/A
Type of implantation, *N* (%)			
Unilateral	35 (64)	35 (64)	N/A
Bilateral	5 (9)	5 (9)	N/A
Bimodal	15 (27)	15 (27)	N/A
Educational level			
VET or lower education (%)	52 (40)	34 (62)	18 (24)
University or higher education (%)	77 (60)	21 (38)	56 (76)
MuRQoL test–retest (%)	95 (74)	37 (67)	58 (78)
Meludia (%)	79 (61)	35 (64)	44 (60)

^a^In case of sequential bilateral implantation, the duration since the first implantation is given

*SD* standard deviation, *N/A* not applicable, *VET* vocational education and training

### Analysis of the sMuRQoL items

Descriptive statistics, discrimination indices and appropriateness of the sMuRQoL items were examined. Each item exhibited suitable values in skewness and kurtosis (Table 2), ranging between ± 1. The discriminative capacity, evaluated according to its presumed dimension, is exceptionally high for each item (D.I. [0.412–0.821]).

**Table 2:** Descriptive statistics, discrimination indices and appropriateness of the items

	Item	Mean	Standard Deviation	Skewness	Kurtosis	Correlation item-test (corrected)	Measure of sampling adequacy (MSA)
Frequency perception	01	3.95	1.018	− 0.974	1.006	0.700	0.908
	02	4.11	0.958	− 0.987	0.589	0.689	0.941
	03	3.86	1.073	− 0.795	− 0.016	0.645	0.895
	04	3.66	0.996	− 0.425	− 0.448	0.674	0.948
	05	3.53	0.936	− 0.509	− 0.012	0.719	0.929
	06	3.44	1.056	− 0.425	− 0.359	0.737	0.947
	07	3.75	1.281	− 0.769	− 0.452	0.821	0.919
	08	3.95	0.883	− 0.532	− 0.399	0.736	0.963
	09	3.53	1.039	− 0.498	− 0.414	0.735	0.940
	10	2.91	1.450	− 0.133	− 1.040	0.648	0.946
	11	3.26	1.399	− 0.659	− 0.361	0.562	0.950
	Total	39.87	9.128	− 0.623	0.112	–	–
Frequency engagement	12	2.85	1.464	0.094	− 1.366	0.662	0.895
	13	3.50	1.294	− 0.459	− 0.941	0.696	0.890
	14	2.95	1.399	− 0.180	− 1.241	0.752	0.888
	15	3.60	1.372	− 0.711	− 0.763	0.732	0.889
	16	2.91	1.240	− 0.270	− 0.927	0.680	0.899
	17	2.56	1.211	0.076	− 1.131	0.620	0.888
	18	2.33	1.251	0.575	− 0.757	0.412	0.851
	Total	20.65	6.971	− 0.185	− 0.912	–	–
Total frequency		60.52	15.028	-0.441	− 0.474	–	–
Importance perception	01	3.32	1.048	− 0.330	− 0.366	0.764	0.918
	02	3.41	1.039	− 0.302	− 0.433	0.748	0.913
	03	3.29	1.130	− 0.425	− 0.406	0.773	0.936
	04	3.47	1.140	− 0.585	− 0.046	0.749	0.891
	05	3.27	1.004	− 0.466	0.072	0.737	0.957
	06	3.49	1.087	− 0.634	0.122	0.746	0.902
	07	3.57	1.151	− 0.788	0.465	0.772	0.887
	08	3.65	0.972	− 0.709	0.291	0.757	0.859
	09	3.45	1.006	− 0.666	0.156	0.674	0.828
	10	2.91	1.309	− 0.250	− 0.597	0.528	0.893
	11	3.28	1.292	− 0.696	0.012	0.532	0.873
	Total	36.88	9.593	− 0.614	0.313	–	–
Importance engagement	12	3.06	1.325	− 0.244	− 0.938	0.670	0.855
	13	3.42	1.178	− 0.509	− 0.313	0.765	0.842
	14	3.20	1.175	− 0.242	− 0.785	0.716	0.853
	15	3.49	1.234	− 0.488	− 0.764	0.708	0.841
	16	3.00	1.193	0.086	− 0.896	0.716	0.880
	17	3.03	1.245	− 0.162	− 0.876	0.627	0.860
	18	2.77	1.319	0.234	− 0.908	0.608	0.891
	Total	21.74	6.878	− 0.256	− 0.218	–	–
Total importance		58.63	15.625	− 0.505	0.194	–	–

### Exploratory factor analysis (EFA)

The internal structure of the sMuRQoL was explored through EFA, conducted separately for the first part of the test—Frequency test—and the second part—Importance test. With respect to the Frequency test, a two-factor structure (perception and engagement) was explored. Both KMO (0.933) and the Bartlett statistic (*p* < 0.001) indicate that the data are suitable for factor analysis. In addition, the MSA (Table 2) for each item was very high, well above the cutoff point of 0.5. The percentage of variance explained by the two factors was 57.54%. The model fit was very good (CFI = 999; RMSR = 0.045), and the factorial loadings were appropriately distributed considering the two theoretical factors (Table 3). Regarding the Importance test, a two-factor structure (perception and engagement) was also explored. Once again, KMO (0.916) and Bartlett (*p* < 0.001) indicated that the data were suitable for conducting the factorial analysis, and the MSA (Table 2) for each item was very high. The percentage of variance explained by the two factors was 59.78% and the model fit was adequate (CFI = 0.999; RMSR = 0.059). Regarding the factorial loadings, they were appropriately distributed, except for item 10, which showed a low factor loading on its reference factor (Table 3). Additionally, in both parts of the test (Frequency and Importance), it would be possible to consider a total score for each, given the high correlation between the factors (frequency: *r* = 0.827; importance: *r* = 0.805).

**Table 3:** Factorial loadings of the items in the exploratory factor analysis

	Frequency test		Importance test	
Item	Factor 1 (perception)	Factor 2 (engagement)	Factor 1 (perception)	Factor 2 (engagement)
01	0.784	− 0.158	0.633	0.066
02	0.815	− 0.102	0.735	0.045
03	0.843	− 0.171	0.719	0.075
04	0.667	0.040	0.738	0.056
05	0.779	− 0.031	0.858	− 0.094
06	0.765	− 0.003	0.938	− 0.147
07	0.736	0.146	0.327	0.527
08	0.842	− 0.088	0.613	0.195
09	0.661	0.111	0.842	− 0.148
10	0.405	0.298	0.089	0.491
11	0.318	0.310	0.231	0.379
12	0.106	0.648	− 0.218	0.994
13	− 0.067	0.821	0.126	0.750
14	− 0.175	0.980	− 0.163	0.889
15	− 0.156	0.919	− 0.084	0.836
16	0.341	0.449	0.193	0.555
17	0.119	0.539	0.277	0.359
18	− 0.033	0.447	0.473	0.205

### Reliability and stability of the scores of sMuRQoL

Regarding the reliability of the sMuRQoL, the internal consistency of the scores across different dimensions of the test was very high, as well as the stability of the scores (test–retest) for the four dimensions, as reflected in the results of the intraclass correlation coefficient *(*Table 4*).*

**Table 4:** Reliability and stability of the Scores of the sMuRQoL

	*n* = 129		*n = *95
	Mcdonald’s omega	Cronbach’s alpha	Intraclass correlation
Frequency			
Overall	0.939	0.940	0.920
Perception	0.920	0.921	0.906
Engagement	0.872	0.879	0.846
Importance			
Overall	0.946	0.945	0.942
Perception	0.925	0.924	0.921
Engagement	0.890	0.887	0.890

### Differences in sMuRQoL scores between CI users and NH individuals

It was observed that NH subjects exhibit significantly higher scores in the Frequency test compared to CI users. Similar to the original study [7], the effect was more pronounced in the perception and the overall frequency subscales than in the engagement subscale. In line with this, no statistically significant differences were found regarding the Importance test, even though participants in the control group exhibited higher scores than those with CI (Table 5).

**Table 5:** Study of differences in scores of the sMuRQoL between Implanted individuals and the Control group

	Control (NH)	Implanted (CI users)		
	*Mean* (SD)	*Mean* (SD)	*t* (*p*)	*d*
Frequency				
Total	68.07 (11.23)	50.36 (13.48)	8.13 (< 0.001)	1.45
Perception	44.36 (6.47)	33.82 (8.72)	7.56 (< 0.001)	1.41
Engagement	23.70 (5.75)	16.55 (6.37)	6.68 (< 0.001)	1.19
Importance				
Total	60.25 (14.21)	56.47 (17.22)	1.35 (0.177)	0.24
Perception	37.66 (8.38)	35.85 (11)	1.05 (0.294)	0.19
Engagement	22.59 (6.64)	20.62 (7.08)	1.62 (0.109)	0.29

*SD *standard deviation, *t s*tudent’s statistic, *d *Cohen’s effect size

### Convergent validity analysis of the sMuRQoL

Regarding the convergent validity of the sMuRQoL in relation to other instruments, MuRQoL scores exhibited positive correlations with scores of Meludia Discovery module tasks on the first attempt for each of its categories (Table 6). Specifically, the Frequency test showed high correlations with all scores on Meludia, particularly standing out in Stable/unstable, Melody and Density.

**Table 6:** Pearson correlations between sMuRQoL scores and scores of Meludia on the first attempt (*n* = 76)

Meludia	Frequency			Importance		
	Total	Perception	Engagement	Total	Perception	Engagement
Rhythm	0.336	0.242	0.383	0.390	0.420	0.299
Spatialization	0.401	0.344	0.387	0.284	0.247	0.303
Stable/unstable	0.557	0.522	0.480	0.279	0.234	0.310
Melody	0.558	0.535	0.470	0.275	0.237	0.296
Density	0.645	0.572	0.584	0.287	0.248	0.304

### Analysis of the discriminative capacity of the instrument regarding musical perception

**Fig. 1:**
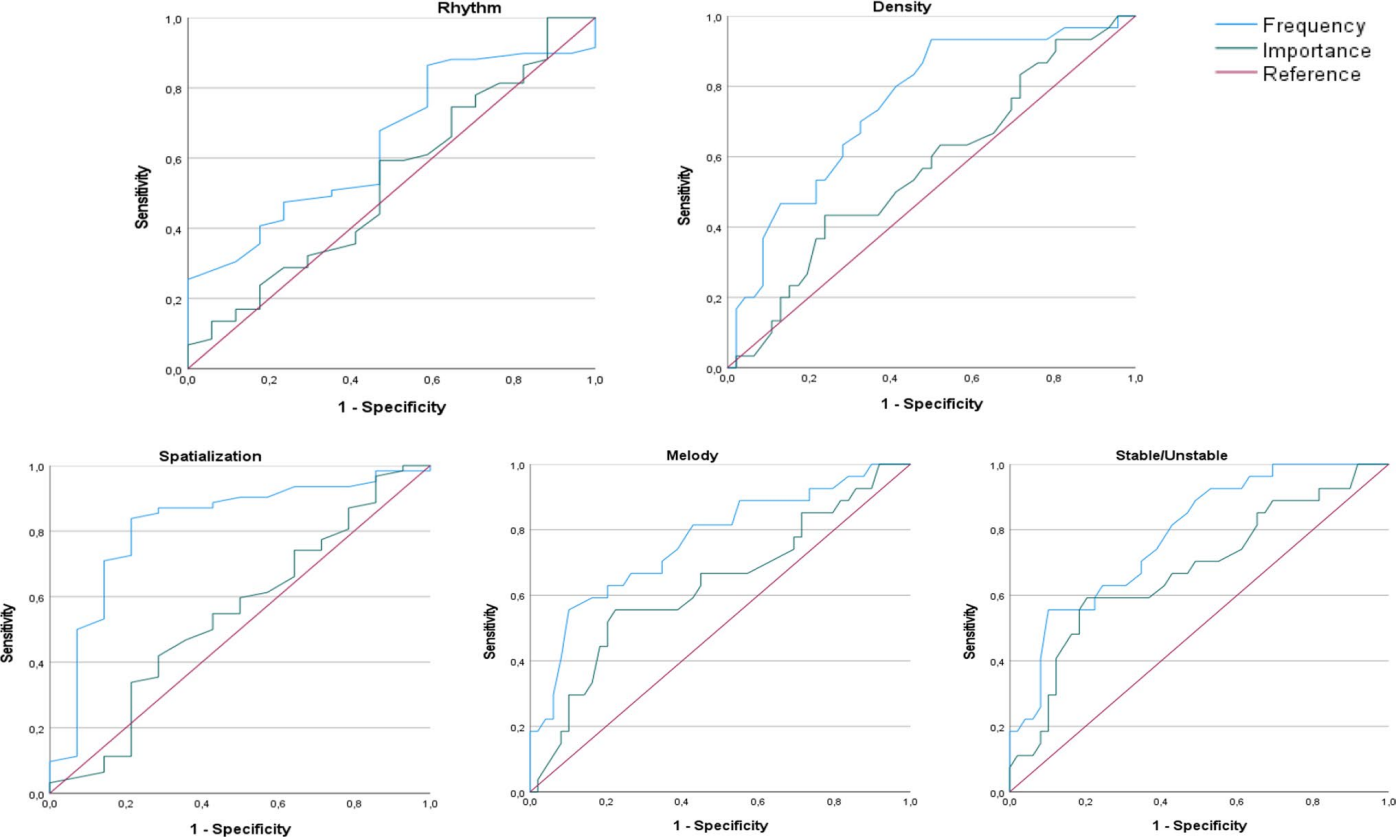
Discriminative capacity of the sMuRQoL based on completing Meludia tasks in a single attempt

The discriminative capacity of sMuRQoL was assessed, considering the completion of the five Meludia Discovery module tasks in a single attempt as the “*Gold Standard”.* The ROC curves are depicted in Fig. 1, calculated using the total scores of Frequency and Importance. Additionally, the area under the curve (AUC) for each Meludia Discovery module task was calculated, showing adequacy across all tasks in relation to the Frequency test. Within these, a notable discriminative capacity was observed, particularly in Spatialization, Stable/Unstable and Density. There was no significant association in Meludia tasks with the Importance test, as expected. (Rhythm: AUC_frequency_ = 0.645; AUC_importance_ = 0.534. Spatialization: AUC_frequency_ = 0.816; AUC_importance_ = 0.547. Stable/Unstable: AUC_frequency_ = 0.783; AUC_importance_ = 0.675. Melody: AUC_frequency_ = 0.764; AUC_importance_ = 0.629. Density: AUC_frequency_ = 0.749; AUC_importance_ = 0.567).

## Discussion

The validation in Turkish and Italian languages of the MuRQoL confirmed the initial findings regarding the reliability and validity of the questionnaire [18, 19]. Our results in 55 CI users and 74 individuals with NH are similar to those obtained in the original MuRQoL and in its two validations in Turkish and Italian, supporting the instrument’s strong cross-cultural validity, as previously reflected in other validations [19].

On the other hand, in the validation of the original MuRQoL [7], the questionnaire’s ability to predict aspects of quality of life was assessed. This was evaluated by demonstrating convergent validity between the MuRQoL Frequency test and the SF12v2 quality of life questionnaire. The SF12v2 is a quality of life questionnaire that can be used to produce utility scores for economic evaluations [7]. In this regard, we aimed to assess the potential convergent validity of the sMuRQoL and its discriminative capacity regarding musical perception using the Meludia tool as a reference. The obtained results have been satisfactory, revealing a positive correlation—showing moderate to high correlations (*r* = 0.24–0.65)—where individuals with high scores in the Frequency Test are capable of achieving better outcomes in Meludia’s Discovery level. This observation aligns with our preliminary findings, suggesting Meludia as a useful tool in assessing and training musical perception. It is noteworthy that these analyses demonstrate the strong discriminative ability of the sMuRQoL instrument—specifically, the Frequency Test—based on good or poor musical perception. However, this questionnaire, being a self-report measure, will never function as a substitute for an objective measure like Meludia, but rather as a complementary tool, cause music training is an intervention aimed at improving auditory perception and processing through various tools of musical activities [9, 10, 13, 17], while the diagnosis of musical perception is a clinical assessment of an individual’s ability to perceive and cognitively process music. The discriminative capacity of the sMuRQoL could prove valuable in clinical practice, as specific results from the AUC of the sMuRQoL Frequency test could be used to establish a cutoff score. This cutoff score would help identify CI users with poorer musical perception, indicating those who would benefit from more extensive musical training. This is particularly relevant since it has been demonstrated that musical training also enhances speech perception, reinforcing the benefits of CI use in other auditory domains [13, 14]. Specifically, in the case of using Meludia, where sessions last at least 1 h, the sMuRQoL would enable us to improve CI users with poorer musical perception selection to optimize available resources.

The MuRQoL questionnaire [7], sound quality assessments [28], and musical perception evaluation platforms like Meludia [17], are some of the suitable tools for assessing the outcomes of music perception in CI users. Therefore, we believe that the sMuRQoL is valuable in evaluating the quality of life in already implanted patients. It serves as a useful tool to discriminate and assist in selecting those CI users who are likely to have a poorer musical perception. In the future, it would be interesting to explore potential differences in questionnaire scores between patients with unilateral or bilateral implantation and those with bimodal implantation. Additionally, an adaptation of the questionnaire for pediatric populations should be considered.

### Limitations

We acknowledge that this study has several limitations inherent to the characteristics of our center and the participant’s sample:

Firstly, the sample of CI users consists solely of patients implanted with a MED-EL System.

Secondly, the age range of the participants is quite broad. Nonetheless, we believe that both groups—CI users and NH—represent highly homogeneous populations with no statistically significant differences between them.

Furthermore, it would be interesting to increase the sample size in future studies. Although the sample size is statistically sufficient for the validation procedure, being a single-center study has limited the sample. In the same sense, the test–retest was carried out with 95 individuals from the total of 129 participants, representing nearly 75% of the sample. The main reason for not applying it to all 129 participants is that those who did not undergo the test–retest did not meet the established temporal criterion, thus preventing the second evaluation from being conducted. However, a sample size of 95 individuals is sufficient to provide evidence of score stability when it accounts for 75% of the total sample obtained.

Finally, there are some inherent limitations of learning and training music listening. Thus, some potential participants declined to participate in the research. This was primarily due to two situations: on one hand, the unpleasant perception of music [5, 6]—in the CI users group—or lack of interest in this field, not perceiving poorer musical perception as a problem—both NH and CI users -; and on the other hand, the completion of Meludia and self-administered online questionnaires require a minimum level of computer literacy, which some elderly participants lacked and were therefore excluded from the study.

## Conclusions

The sMuRQoL is a reliable instrument with adequate evidence of validity based on internal structure. The present study offers an accessible, low-cost, and quickly executable tool in Spanish. The sMuRQoL has proven to be a reliable instrument in discriminating among patients with greater difficulties in musical perception. Therefore, it constitutes a valuable tool for selecting individuals within CI users who might benefit from musical training platforms such as Meludia.

## Data Availability

The data presented in this study are available on request from the corresponding author.

## References

[1] Ma C, Fried J, Nguyen SA, Schvartz-Leyzac KC, Camposeo EL, Meyer TA, Dubno JR, McRackan TR (2023) Longitudinal speech recognition changes after cochlear implant: systematic review and meta-analysis. Laryngoscope 133:1014–1024. 10.1002/lary.30354 36004817 10.1002/lary.30354

[2] Limb CJ, Roy AT (2014) Technological, biological, and acoustical constraints to music perception in cochlear implant users. Hear Res 308:13–26. 10.1016/j.heares.2013.04.009 23665130 10.1016/j.heares.2013.04.009

[3] Wheeler HJ, Hatch DR, Moody-Antonio SA, Nie Y (2022) Music and speech perception in prelingually deafened young listeners with cochlear implants: a preliminary study using sung speech. J Speech Lang Hear Res 65:3951–3965. 10.1044/2022_JSLHR-21-00271 36179251 10.1044/2022_JSLHR-21-00271

[4] Driscoll V, Gfeller K, Tan X, See RL, Cheng HY, Kanemitsu M (2015) Family involvement in music impacts participation of children with cochlear implants in music education and music activities. Cochlear Implants Int 16:137–146. 10.1179/1754762814Y.0000000103 25431978 10.1179/1754762814Y.0000000103 PMC4420640

[5] Gfeller K, Driscoll V, Schwalje A (2019) Adult cochlear implant recipients’ perspectives on experiences with music in everyday life: a multifaceted and dynamic phenomenon. Front Neurosci 13:1229. 10.3389/fnins.2019.01229 31824240 10.3389/fnins.2019.01229 PMC6882382

[6] Lassaletta L, Castro A, Bastarrica M, Pérez-Mora R, Herrán B, Sanz L, de Sarriá MJ, Gavilán J (2008) Changes in listening habits and quality of musical sound after cochlear implantation. Otolaryngol Head Neck Surg 138:363–367. 10.1016/j.otohns.2007.11.028 18312886 10.1016/j.otohns.2007.11.028

[7] Dritsakis G, van Besouw RM, Kitterick P, Verschuur CA (2017) A music-related quality of life measure to guide music rehabilitation for adult cochlear implant users. Am J Audiol 26:268–282. 10.1044/2017_AJA-16-0120 28614845 10.1044/2017_AJA-16-0120

[8] Lassaletta L, Castro A, Bastarrica M, Pérez-Mora R, Madero R, De Sarriá J, Gavilán J (2007) Does music perception have an impact on quality of life following cochlear implantation? Acta Otolaryngol 127:682–686. 10.1080/00016480601002112 17573562 10.1080/00016480601002112

[9] Peretz I, Gosselin N, Nan Y, Caron-Caplette E, Trehub SE, Béland R (2013) A novel tool for evaluating children’s musical abilities across age and culture. Front Syst Neurosci 7:30. 10.3389/fnsys.2013.00030 23847479 10.3389/fnsys.2013.00030 PMC3707384

[10] Fuller CD, Galvin JJ, Maat B, Başkent D, Free RH (2018) Comparison of two music training approaches on music and speech perception in cochlear implant users. Trends Hear 22:2331216518765379. 10.1177/2331216518765379 29621947 10.1177/2331216518765379 PMC5894911

[11] Jiam NT, Deroche ML, Jiradejvong P, Limb CJ (2019) A randomized controlled crossover study of the impact of online music training on pitch and timbre perception in cochlear implant users. J Assoc Res Otolaryngol 20:247–262. 10.1007/s10162-018-00704-0 30815761 10.1007/s10162-018-00704-0 PMC6514036

[12] Firestone GM, McGuire K, Liang C, Zhang N, Blankenship CM, Xiang J, Zhang F (2020) A preliminary study of the effects of attentive music listening on cochlear implant users’ speech perception, quality of life, and behavioral and objective measures of frequency change detection. Front Hum Neurosci 14:110. 10.3389/fnhum.2020.00110 32296318 10.3389/fnhum.2020.00110 PMC7136537

[13] Smith L, Bartel L, Joglekar S, Chen J (2017) Musical rehabilitation in adult cochlear implant recipients with a self-administered software. Otol Neurotol 38:e262–e267. 10.1097/MAO.0000000000001447 28806336 10.1097/MAO.0000000000001447

[14] Torppa R, Huotilainen M (2019) Why and how music can be used to rehabilitate and develop speech and language skills in hearing-impaired children. Hear Res 380:108–122. 10.1016/j.heares.2019.06.003 31265971 10.1016/j.heares.2019.06.003

[15] Pastor EJ (2016) Rehabilitación en implantes cocleares. Rev Med Clin Conde 27:834–839. 10.1016/j.rmclc.2016.11.015 10.1016/j.rmclc.2016.11.015

[16] Hwa TP, Wen CZ, Ruckenstein MJ (2021) Assessment of music experience after cochlear implantation: a review of current tools and their utilization. World J Otorhinolaryngol Head Neck Surg 7:116–125. 10.1016/j.wjorl.2021.02.003 33997721 10.1016/j.wjorl.2021.02.003 PMC8103528

[17] Calvino M, Zuazua A, Sanchez-Cuadrado I, Gavilán J, Mancheño M, Arroyo H, Lassaletta L (2024) Meludia platform as a tool to evaluate music perception in pediatric and adult cochlear implant users. Eur Arch Otorhinolaryngol 281:629–638. 10.1007/s00405-023-08121-7 37480418 10.1007/s00405-023-08121-7 PMC10796694

[18] Akbulut AA, Çiprut A, Akdeniz E, Batman Ç (2022) Translation and validation of the music-related quality of life questionnaire for adults with cochlear implant in Turkish language. Eur Arch Otorhinolaryngol 279:685–693. 10.1007/s00405-021-06693-w 33599840 10.1007/s00405-021-06693-w

[19] Frosolini A, Parrino D, Mancuso A, Coppola N, Genovese E, de Filippis C (2022) The music-related quality of life: Italian validation of MuRQoL into MUSQUAV questionnaire and preliminary data from a cohort of postlingually deafened cochlear implant users. Eur Arch Otorhinolaryngol 279:4769–4778. 10.1007/s00405-022-07258-1 35089391 10.1007/s00405-022-07258-1 PMC9474524

[20] Allen MJ, Yen WM (1979) Introduction to measurement theory. Brooks/Cole Pub. Co, Monterey, California

[21] MELUDIA (2022) https://www.meludia.com/es/. Accessed 17 Nov 2023

[22] World Medical Association (2013) Declaration of Helsinki: ethical principles for medical research involving human subjects. JAMA 310:2191. 10.1001/jama.2013.281053 24141714 10.1001/jama.2013.281053

[23] Muñiz J, Fonseca-Pedrero E (2019) Diez pasos para la construcción de un test. Psicothema 7–16. 10.7334/psicothema2018.291

[24] Ferrando PJ, Lorenzo-Seva U, Hernández-Dorado A, Muñiz J (2022) Decálogo para el análisis factorial de los ítems de un test. Psicothema 7–17. 10.7334/psicothema2021.456 10.7334/psicothema2021.456 35048890

[25] Hu L, Bentler PM (1999) Cutoff criteria for fit indexes in covariance structure analysis: conventional criteria versus new alternatives. Struct Equ Modeling 6:1–55. 10.1080/10705519909540118 10.1080/10705519909540118

[26] Cohen J (1988) Statistical power analysis for the behavioral sciences. 2nd edn. L. Erlbaum Associates, Hillsdale, New Jersey

[27] FACTOR (2023) https://psico.fcep.urv.cat/utilitats/factor/Download.html. Accessed 17 Nov 2023.

[28] Calvino M, Gavilán J, Sánchez-Cuadrado I, Pérez-Mora RM, Muñoz E, Díez-Sebastián J, Lassaletta L (2016) Using the HISQUI29 to assess the sound quality levels of Spanish adults with unilateral cochlear implants and no contralateral hearing. Eur Arch Otorhinolaryngol 273:2343–2353. 10.1007/s00405-015-3789-0 26440105 10.1007/s00405-015-3789-0

